# A chance-constrained stochastic approach to intermodal container routing problems

**DOI:** 10.1371/journal.pone.0192275

**Published:** 2018-02-13

**Authors:** Yi Zhao, Ronghui Liu, Xi Zhang, Anthony Whiteing

**Affiliations:** 1 School of traffic and transportation Beijing Jiaotong University, Beijing, China; 2 Institute for Transport Studies, University of Leeds, Leeds, United Kingdom; Rutgers The State University of New Jersey, UNITED STATES

## Abstract

We consider a container routing problem with stochastic time variables in a sea-rail intermodal transportation system. The problem is formulated as a binary integer chance-constrained programming model including stochastic travel times and stochastic transfer time, with the objective of minimising the expected total cost. Two chance constraints are proposed to ensure that the container service satisfies ship fulfilment and cargo on-time delivery with pre-specified probabilities. A hybrid heuristic algorithm is employed to solve the binary integer chance-constrained programming model. Two case studies are conducted to demonstrate the feasibility of the proposed model and to analyse the impact of stochastic variables and chance-constraints on the optimal solution and total cost.

## Introduction

In recent decades, international trade has been growing rapidly throughout the world. At the same time, the development of intermodal logistics has offered opportunities for freight companies to replan their operations. The Intermodal freight transportation (IFT), defined as the transportation of cargoes by two or more different modes of transport [1–2], is a relatively young domain [3]. Generally, there are five key research issues on intermodal transportation as summarised in [4].

(i) *Intermodal transportation policy*: to analyse the effect of different policies on intermodal transportation, such as tax changes, train speed changes, train length changes, service quality changes and new infrastructure investment.
(ii) *Intermodal network design*: to address how to construct or improve intermodal transportation infrastructure in order to satisfy the demands and optimize the objective criteria [5].
(iii) *Intermodal service design*: to decide on issues such as service frequency and terminal operation [6].
(iv) *Intermodal routing problem*: to specify the routes to transport each demand from its origin to its destination through an intermodal network.
(v) *Empty container repositioning*: to optimize the reposition plan in order to meet forecast demand and to minimise repositioning costs.

This paper focuses on issue (*iv*), i.e. the intermodal freight routing problem, and more specifically the intermodal cargo routing problem. As a cargo loading unit usually refers to a standardized container measured in Twenty-foot Equivalent Units (TEU), we limit our study scope to the intermodal container routing problem (ICRP) in this paper. The ICRP is described as the need to specify the container route that minimises the total transportation cost while satisfying delivery time constraints for each container freight demand. Table 1 lists the key literature on ICRP in terms of the stochasticity represented. The pioneering work of Barnhart and Ratliff [7] considered an ICRP which involves trailers and containers in a truck and rail IFT system. They formulated the ICRP as a shortest path problem minimising the sum of transportation cost and inventory cost and solved it by the weighted b-matching algorithm.

**Table 1:** Overview of intermodal container routing problem.

Problems	Stochastic travel time	Stochastic transfer time	Stochastic demand	Key literature
Classical deterministic ICRP	No	No	No	Ayar and Yaman [10]; Barnhart and Ratliff [7]; Chang [9]; Cho et al. [8]; Kim et al. [18]; Moccia et al. [22]; Ziliaskopoulos and Wardell [11]
Combined ICRP	No	No	No	Crainic et al. [16]; Crainic et al. [17]; Meng et al. [15]; Riessen et al. [19]
Stochastic ICRP	Yes	No	No	Min [13]
Stochastic Combined ICRP	Yes	No	Yes	Demir et al. [14]
Stochastic ICRP	Yes	Yes	No	**This work**

The literatures that followed can be broadly classified as single-objective ICRP and multi-objective ICRP. The single-objective ICRP is usually to minimise the total cost, while the multi-objective ICRP is usually to minimise both transportation cost and travel time [8] or the weighted sum of the two [9]. For single-objective ICRPs, Ayar and Yaman [10] formulated the ICRP in a truck-ship intermodal network as a multi-commodity routing problem with a single objective to minimise the sum of transportation cost and inventory cost, where the transportation modes and routes were determined for each commodity. Ziliaskopoulos and Wardell [11] proposed a label correcting algorithm (i.e. scanning an eligible list) to find the least-time paths from origin nodes to destination nodes in a time-dependent intermodal network, with the objective to minimise the total travel time. The model was tested on several realistic networks with 50, 100, 500 and 1000 nodes, respectively. For multi-objective ICRPs, Cho et al. [8] formulated an integer programming model which considered export and import containers simultaneously. A label setting algorithm was applied to get the Pareto optimal solutions, and the model was demonstrated in a large-scale ship-railway-air network between Busan and Rotterdam. Chang [9] considered a container routing problem for minimising transportation cost and travel time in a truck-ship-air intermodal network, and formulated a multi-commodity flow problem with time window constraint, where each commodity represented a single origin-destination demand. The problem was formulated as a mixed integer nonlinear programming model and a heuristic algorithm, with relaxation and decomposition is proposed to solve the problem.

In addition to travel time and cost, other factors have been taken into account as objectives in ICRP, such as travel time variability [12], risk [13] and CO_2_ emission [14]. The ICRPs were also combined with other relevant IFT problems, such as empty container reposition [15] and service network design [16–18]. Meng et al. [15] developed a mixed integer linear programming model for the routing of both laden containers and empty containers. Besides, the model determined the number of empty containers loaded, unloaded and transhipped at seaports simultaneously in an inland-maritime network. Crainic et al. [16] established a mixed integer nonlinear programming model to solve the problems of service design, freight routing and terminal policies with decomposition and column generation principles. An extension of this work was presented by Crainic et al. [17], who formulated an integer nonlinear programming to assign multi-commodity freight to a multimodal transportation network taking into consideration the operating cost, delay cost and energy consumption. Kim et al. [18] constructed a mixed integer programming to determine the transportation flow quantity and transportation mode on each route in a truck-rail intermodal network in Korea. The objective was to minimise the system cost with the limitation of cargo volumes at seaports and number of vehicles at each mode. Riessen et al. [19] combined a path based formulation and a minimum flow network formulation to design a service network in Europe taking into consideration both self-operated service and subcontracted service, with specified routes of freight.

All the above literature considered ICRP under deterministic conditions, where travel times and transfer times were treated as deterministic and represented an average condition. In practice, however, container transportation is full of unpredictable and stochastic elements. For example, Li et al. [20] described the uncertainty in the planning of transit itinerary and proposed a two-phase approach to find the best transit itineraries under uncertainty. Meng et al. [3] reviewed the routing and scheduling problem in container maritime transportation and concluded there are many uncertainties in container transportation. Travel time is perhaps the most uncertain factor in intermodal container transportation. For example, in the railway freight system, due to the lower priority of freight transportation, the travel time of freight trains is frequently impacted by passenger trains. As a result, it is difficult to ensure the punctuality of freight trains. While in the maritime system, travel time is also stochastic because of unexpected weather conditions, for example. In addition, container transfer time can also be variable due to different employee productivity. Such variabilities in an IFT system directly affect the chances of achieving good service connections and on-time delivery, and as such are a major concern for freight companies. Therefore, it is essential to consider the stochasticity of travel time and transfer time in container transportation, especially in a rail-sea transportation system.

So far, there has been limited research on this issue, as noted in Demir et al. [14], Min [13], and Dong [21]. Specifically, Demir et al. [14] constructed a mixed integer nonlinear programming model for a green intermodal service network design problem with time uncertainty and demand uncertainty. A sample average approximation method was applied to solve the problem and minimise the total cost including transportation cost, transhipment cost, delayed cost and CO_2_ emissions-related cost. In Min [13], a chance-constrained goal programming was constructed to select the best intermodal route in order to minimise cost and risk as well as satisfy on-time delivery requirement. Dong [21] presented a two stage stochastic programming model to deal with both service capacity planning and container routing, taking into account uncertain demand. A Progressive Hedging Algorithm (PHA) was employed to solve the problem.

High efficiency algorithms are crucial for generating a practical container routing plan. There has been extensive research on exact algorithms, such as branch and bound algorithms, cutting-plane methods, dynamic programming algorithms [8] and column generation algorithms [16]. However, as the container routing problem in real life is NP-hard, exact algorithms cannot compute the optimal solution efficiently and thus approximate algorithms are called for. Heuristic algorithms have been developed by Chang, Dong, Meng et al. and Moccia et al [9, 15, 21–22]. Some of these algorithms are combined with the decomposition technique, such as Lagrangian relaxation and Progressive hedging algorithm (PHA). In Chang [9], Lagrangian relaxation was used to separate the initial problem into two sub-problems which were solved by different exact algorithms. A Progressive hedging algorithm (PHA) was employed by Dong [21] to decompose large scale problems. Besides, there are also other approximation algorithms such as Sample Average Approximation [14] and comprehensive evaluation methods [12]. For example, Yang et al. [12] presented an intermodal network optimization model based on goal programming to evaluate the objective value of 36 alternative routes from China to India considering transportation cost, transit time and travel time variability. The model was tested on a real intermodal network with two Chinese origins and four Indian destinations.

In intermodal container transportation, the transportation time consists of travel time by different modes and transfer time between different modes, which can both affect the probability of on-time delivery and the feasibility of container route plan. Thus the complexity of robust container route plan lies in taking into account stochastic travel time and stochastic transfer time simultaneously. However, most literatures only consider one of them.

In response to the complexity, this paper presents a chance constrained programming to deal with the container routing problem with respect to laden export container cargo and stochastic time parameters in an intermodal sea-rail network. The aim is to select the best routes for each container demand transported from its origin to its destination by rail and sea. The travel time and transfer time are treated as random variables. The proposed methodology will be demonstrated by one small-scale case and one practical sized case respectively, where both the deterministic situation and the stochastic situation are considered. The effects of stochastic time parameters on the optimal solution, and the relationships between cost and stochasticity, are also discussed. The contribution of this paper is threefold: (1) formulating a chance-constrained programming problem for the ICRP with stochastic travel times and stochastic transfer time; (2) proposing a hybrid heuristic algorithm to solve the problem; and (3) examining the effects of stochastic variables on different types of system costs and on cargo delivery punctuality.

The rest of this paper is organised as follows. *Section Problem statement* introduces the container routing problem with stochastic time variables. *Section A dual chance-constrained ICRP with stochastic time variables* formulates the binary integer chance-constrained programming model for the ICRP with stochasticity. *Section A hybrid heuristic solution algorithm* describes the proposed hybrid solution method, while *Section Numerical example* presents a numerical example to illustrate the workings of the model and conducts sensitivity tests on the model parameters. Finally, *Section Conclusion* summaries the findings and highlights future research directions.

## Problem statement

Intermodal container routing in a sea-rail network is complicated by three characteristics. First, Compared with other bulk freight transportation, containerized traffic is often more time-sensitive: goods in the container may be perishable or consumer goods with a short life cycle. Hence the delivery time of each demand is an important consideration in the routing problem. This requires simultaneous minimization of travel cost and on-time arrival with a predetermined punctuality. Second, in a sea-rail intermodal system, the transportation services usually follow fixed schedules and are less flexible than road services. Thus, the punctuality of services becomes extremely important. Unexpected delay on railway or at transfer sea ports may lead to missing scheduled sea sailings which depart according to fixed schedules. Third, Because of the transhipment between trains and ships, the component costs of intermodal container transportation include not only the transportation costs, but also inventory and transfer costs. The container routing problem addressed in this research is to select the minimal-cost routes considering stochastic travel time variables for total container demand originating from multiple inland railway stations and destining to a single foreign seaport in a sea-rail network.

**Fig 1:**
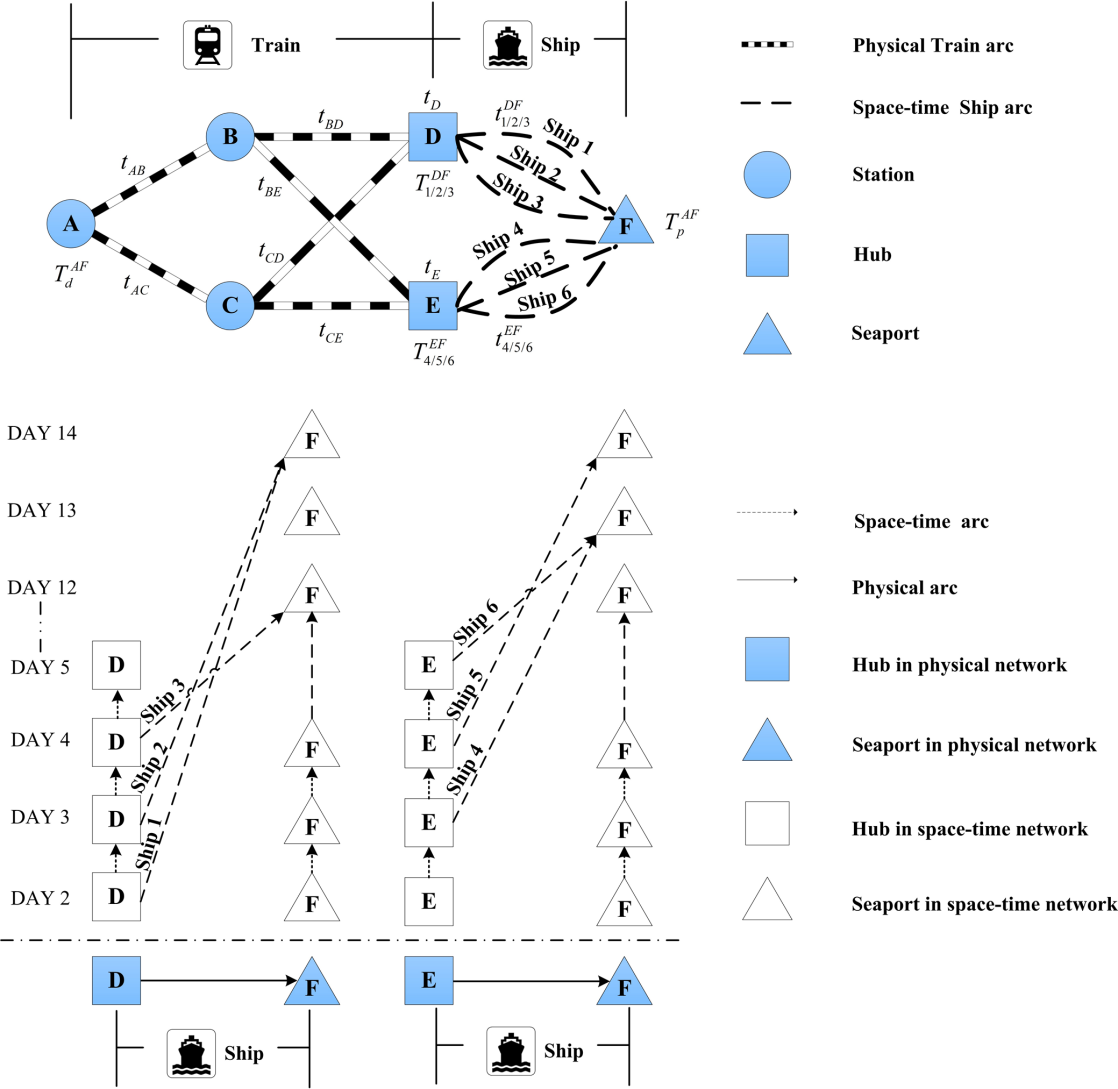
An illustration of a rail-sea intermodal freight transportation network.

To illustrate the problem, we first consider the case from a single origin to a single destination. Fig 1 illustrates a sea-rail network with a single origin *A*, two rail loading locations *B* and *C*, two hubs *D* and *E* where container cargoes can transfer between different modes, and one destination *F*.

An export container cargo from *A* to *F* (or *AF* for short) can take the following twelve possible routes.

$$\begin{array}{l} A\overset{Train}{\to }B\overset{Train}{\to }D\overset{Ship(1/2/3)}{\to }F\\ A\overset{Train}{\to }C\overset{Train}{\to }D\overset{Ship(1/2/3)}{\to }F\\ A\overset{Train}{\to }B\overset{Train}{\to }E\overset{Ship(4/5/6)}{\to }F\\ A\overset{Train}{\to }C\overset{Train}{\to }E\overset{Ship(4/5/6)}{\to }F \end{array}$$

For each container demand, there is a scheduled departure time at the origin railway station, and a promised delivery time at the destination port. Early departure is forbidden while late delivery is allowed but incurs penalty. For demand *AF*, assuming the optimal route is

$$A\overset{Train}{\to }C\overset{Train}{\to }E\overset{Ship4}{\to }F$$

Let $T_{d}^{AF}$ denote the train departure time at origin *A*, *t*_*AC*_ and *t*_*CE*_ respectively the travel time on rail links *AC* and *CE*, *t*_*E*_ the transfer time at port *E*, and $T_{4}^{EF}$ the scheduled departure time of ship 4 from port *E*. In deterministic circumstance, the constraint below should be satisfied to ensure that the container demand *AF* can catch the ship.

$$T_{d}^{AF}+t_{AC}+t_{CE}+t_{E}\leq T_{4}^{EF}$$ (1)

Such constraint could be violated when the train travel times and transfer time are uncertain. In this paper, the travel time by train/ship and the transfer time at hubs are defined as random variables which follow normal distribution and uniform distribution, respectively. Assuming all the ships depart on time according to fixed schedules, the two chance constraints in Eqs (2)–(3) based on stochastic time variables are considered for cargo demand *AF*.

Eq (2) sets out that the required probability that cargo *AF* can board the ship before the scheduled departure is higher than a given confidence level *α*. Eq (3) requires a confidence level by which the cargo *AF* arrives at the destination port by the promised delivery time no lower than *β*, where $T_{p}^{AF}$ is the promised delivery time.

$$\mathrm{Pr}\left\{T_{d}^{AF}+t_{AC}+t_{CE}+t_{E}\leq T_{4}^{EF}\right\}\geq \alpha$$ (2)

$$\mathrm{Pr}\left\{T_{d}^{AF}+t_{AC}+t_{CE}+t_{E}+t_{4}^{EF}\leq T_{p}^{AF}\right\}\geq \beta$$ (3)

Based on the above constraints, a chance constrained programming of ICRP is formulated, which takes into account stochastic travel times on the railway and at sea, as well as stochastic transfer time at hubs. We present the model formulation in *Section A dual chance-constrained ICRP with stochastic time variables* below and a solution algorithm based on Monte-Carlo simulation in combination with a neural network in *Section A hybrid heuristic solution algorithm*.

## A dual chance-constrained ICRP with stochastic time variables

In stochastic optimization, there are broadly three types of stochastic programming methods: expected value models, chance constrained programming, and dependent chance programming [23]. The chance constrained programming was first proposed in Charnes and Cooper [24] to solve optimization problems under various uncertain circumstances and to ensure that the decisions meet certain constraints with certain levels. The model has been applied widely in different subject areas, such as in biology [25], finance [26] and transportation [27].

In this paper, we formulate our stochastic ICRP problem as a chance-constrained optimisation model, in the sea-rail intermodal network. The sea-rail intermodal network is modelled as a directed graph *G = (N*, *A)*. The set *N* of nodes represents the railway stations, transfer hubs and seaports, while the set *A* of arcs denotes railway arcs and ship arcs. Before proposing the chance constrained programming model for the container routing problem with stochastic parameters, we define the notations to be used as shown in Table 2.

### Notations

**Table 2:** Notations.

**Index and sets**	
*N*	the set of nodes, including railway stations, transfer hubs and seaports
*A*	the set of arcs, including railway arcs and ship arcs
*O*	the set of origin stations
*H*	the set of transfer hubs, where the cargo is transferred from trains to ships
*D*	the set of destination ports
*S*	the set of ships scheduled from the transfer hubs to the destination ports
*i*, *j*	two nodes except destination ports, *i*, *j* ∈ *N*\*D*
*a*	a railway link, *a* ∈ *A*
*o*	an origin station, *o* ∈ *O*
*h*	an intermodal transfer hub, *h* ∈ *H*
*d*	a destination port, *d* ∈ *D*
*s*	a ship service, *s* ∈ *S*
*k*	a candidate railway route, *k* ∈ *R*^*od*^. *R*^*od*^ is the set of *k* shortest routes (based on travel distance) for cargo from origin *o* to destination *d* (*od* for short)
**Input parameters**	
*q*^*od*^	the transportation volume of the cargo *od* (TEUs)
$T_{d}^{od}$	the departure time for the cargo *od* (hours)
$T_{p}^{od}$	the promised arrival time for the cargo *od* (hours)
$T_{s}^{hd}$	the scheduled departure time of ship *s* from *h* to *d* (days)
*c*_*a*_	the unit cost of transportation on railway link *a* (US$/TEU)
$c_{s}^{hd}$	the unit cost of transportation by ship *s* between seaports *h* and *d* (US$/TEU)
*c*_*h*_	the unit cost of transfer at *h* (US$/TEU)
*c*_*I*_	the unit cost of inventory (US$/TEU/day)
$c_{p}^{1}$	the late-delivery penalty cost (US$/TEU/day)
$c_{p}^{2}$	the unfulfilled demand penalty cost (US$/TEU)
*b*_*a*_	the capacity of railway link *a* (TEUs)
$b_{s}^{hd}$	the capacity of ship *s* from *h* to *d* (TEUs)
*b*_*h*_	the capacity of transfer at *h* (TEUs)
**Auxiliary variables**	
*q*_*a*_	the transportation volume on railway link *a* (TEUs)
$q_{s}^{hd}$	the transportation volume on ship *s* from *h* to *d* (TEUs)
$t_{s}^{hd}$	the transportation time from hub *h* to destination port *d* by ship *s* (days)
*t*_*a*_	the transportation time on railway link *a* (hours)
*t*_*h*_	the transfer time at hub *h* (hours)
$t_{k}^{od}$	the transportation time of the railway route *k* for cargo *od* (hours)
**Decision variables**	
$x_{k}^{od}$	a binary variable, equal to 1 if route *k* is selected as the optimal route for the cargo *od* in railway network; 0 otherwise
$y_{h}^{od}$	a binary variable, equal to 1 if the hub *h* is selected as the transfer from cargo *od*; 0 otherwise
$z_{hs}^{od}$	a binary variable, equal to 1 if the cargo *od* is transported by ship *s* from *h* to port *d*; 0 otherwise
**Indicator variables**	
$\delta_{a,k}^{od}$	a binary variable equal to 1 if the link *a* is on the railway route *k* of the cargo *od* in the railway network; 0 otherwise
*η*^*od*^	a binary variable equal to 1 if the cargo *od* catches the succeeding ship service, i.e. $T_{d}^{od}+\sum_{k\in R^{od}}x_{k}^{od}t_{k}^{od}+\sum_{h\in H}y_{h}^{od}t_{h}-\sum_{h\in H}\sum_{s\in S}z_{hs}^{od}T_{s}^{hd}\leq 0$; 0 otherwise
*γ*^*od*^	a sign function of the difference between the promised delivery time and actual delivery time for cargo *od*, i.e. $\gamma^{od}=\mathrm{sgn}\left(\sum_{h\in H}\sum_{s\in S}z_{hs}^{od}\left(T_{s}^{hd}+t_{s}^{hd}\right)-T_{p}^{od}\right)$, where $\mathrm{sgn}\left(x\right)=\{\begin{array}{c} -1,\;x<0\\ 0,\;x=0\\ 1,\;x>0 \end{array}$

### A chance-constrained optimisation formulation

Our problem is formulated based on the following assumptions:

Assumption 1: The cargo demands are represented in TEU.
Assumption 2: All containers depart from their origins on time.
Assumption 3: A container missing its succeeding ship service is treated as an unfulfilled demand and incurs a fixed penalty cost.
Assumption 4: The railway transportation cost on arcs per container is proportional to the arc length.
Assumption 5: The travel time *t*_*a*_ on a railway link *a* follows a normal distribution [28], i.e. *t*_*a*_ ∼ *N*(*μ*_*a*_, *σ*_*a*_) with *μ*_*a*_ the mean travel time and $\sigma_{a}=f\sigma_{a}^{0}$ the standard deviation on link *a*, where $\sigma_{a}^{0}=1$ *hour* and *f* is a multiplier. The sea travel time $t_{s}^{hd}$ by ship *s* also follows a normal distribution, i.e. $t_{s}^{hd}∼N\left(\mu_{s},\sigma_{s}\right)$ with *μ*_*s*_ the mean and $\sigma_{s}=f\sigma_{s}^{0}$ standard deviation, where $\sigma_{s}^{0}=1$ *day* and *f* is a multiplier [29].
Assumption 6: The transfer time at a hub *h* follows a uniform distribution [30], i.e. *t*_*h*_ ∼ *U*(*m*_*h*_, *n*_*h*_), where *m*_*h*_ and *n*_*h*_ denote the minimum and maximum values of the distribution respectively.

Assumption 1 and Assumption 2 are common to model the characteristic of container demand in the literature on ICRP. Assumption 3 ensures that the container missing ship service is modelled, which leads to a penalty cost as a part of operation cost. Assumption 4 is in line with railway transportation practice that unit transportation cost is fixed and proportion to travel distance. For railway transportation, there are two types of delay. One is that trains are affected directly by some reasons, such as bad weather, staff operation mistakes, dispatching mistakes and infrastructure reconstruction. The other one is that trains are affected by other delayed trains. Both the two delays can result in stochastic travel time. For maritime transportation, the total cruise time consists of port service time and travel time between ports, which can be highly variable due to congestion, handling operation and adverse weather. Therefore, Assumption 5 describes the travel times by rail and sea as random variables following different normal distribution. Assumption 6 describes the transfer time as a random variable following a uniform distribution to model uncertainty at hubs, e.g., congestion, disruption and handling.

The objective function is the expected value of total cost which contains five elements: transportation cost, transfer cost, inventory cost, late delivery penalty cost and non-fulfilment penalty cost. For each unit of demand between origin *o* and destination *d*, these costs are represented as follows.

(i) Transportation cost:

$$\sum_{k\in R^{od}}\sum_{a\in A}c_{a}x_{k}^{od}\delta_{a,k}^{od}+\sum_{h\in H}\sum_{s\in S}c_{s}^{hd}z_{hs}^{od},o\in O,d\in D$$ (4)

In Eq (4), the first term represents the rail transportation cost while the second term is the maritime transportation cost.

(ii) Transfer cost:

$$\sum_{h\in H}c_{h}y_{h}^{od},o\in O,d\in D$$ (5)

Based on the characteristics of intermodal container transportation, the container cargoes need to be transferred between different modes at transfer hubs. Thus, the transfer cost in Eq (5) is incurred by moving all container demands from rail terminals to seaports as well as loading and unloading operation.

(iii) Inventory cost:

$$\begin{array}{l} \eta^{od}c_{I}(\sum_{h\in H}\sum_{s\in S}z_{hs}^{od}T_{s}^{hd}-T_{d}^{od}-\sum_{k\in R^{od}}x_{k}^{od}t_{k}^{od}-\sum_{h\in H}y_{h}^{od}t_{h}\\ \;+\min\left(\gamma^{od},0\right)\left(\sum_{h\in H}\sum_{s\in S}z_{hs}^{od}\left(T_{s}^{hd}+t_{s}^{hd}\right)-T_{p}^{od}\right)),o\in O,d\in D \end{array}$$ (6)

Inventory cost is incurred by containers waiting to be transferred or picked up. In the model, we only consider the inventory cost in transfer hubs (as in the first term) and destination ports (as in the second term).

(iv) Late delivery penalty cost:

$$\eta^{od}c_{p}^{1}\max\left(\gamma^{od},0\right)\left(\sum_{h\in H}\sum_{s\in S}z_{hs}^{od}\left(T_{s}^{hd}+t_{s}^{hd}\right)-T_{p}^{od}\right),o\in O,d\in D$$ (7)

Due to the uncertain travel time, the delivery time for each container cargo is stochastic. A delayed cargo will result in late delivery penalty cost which is proportional to delay time, as given in Eq (7).

(v) Non-fulfilment penalty cost:

$$c_{p}^{2}\left(1-\eta^{od}\right),o\in O,d\in D$$ (8)

Due to the delay caused by stochastic travel time and stochastic transfer time, some cargoes would fail to arrive at transfer hubs for transhipment. These unsatisfied demands will cause the non-fulfilment penalty cost as shown in Eq (8).

The total cost *C*^*od*^ for each unit of demand is then the sum of these six elements:

$$\begin{array}{l} C^{od}=\sum_{a\in A}c_{a}x_{k}^{od}\delta_{a,k}^{od}+\sum_{h\in H}\sum_{s\in S}c_{s}^{hd}z_{hs}^{od}\\ \;+\sum_{h\in H}c_{h}y_{h}^{od}\\ \;+\eta^{od}c_{I}\left(\sum_{h\in H}\sum_{s\in S}z_{hs}^{od}T_{s}^{hd}-T_{d}^{od}-\sum_{k\in R^{od}}x_{k}^{od}t_{k}^{od}-\sum_{h\in H}y_{h}^{od}t_{h}\right)\\ \;+\eta^{od}c_{I}\min\left(\gamma^{od},0\right)\left(\sum_{h\in H}\sum_{s\in S}z_{hs}^{od}(T_{s}^{hd}+t_{s}^{hd})-T_{p}^{od}\right)\\ \;+\eta^{od}c_{p}^{1}\max(\gamma^{od},0)\left(\sum_{h\in H}\sum_{s\in S}z_{hs}^{od}(T_{s}^{hd}+t_{s}^{hd})-T_{p}^{od}\right)\\ \;+c_{p}^{2}\left(1-\eta^{od}\right) \end{array}$$ (9)

Our stochastic ICRP is then to minimise the total expected cost:

$$\min\sum_{o\in O,d\in D}q^{od}E[C^{od}]$$ (10)

subject to the following constraints:

(a) *Flow conservation constraint*:

$$\sum_{o\in O,d\in D}q^{od}\sum_{k\in R^{od}}x_{k}^{od}\delta_{a,k}^{od}=q_{a},a\in A$$ (11)

$$\sum_{o\in O}q^{od}z_{hs}^{od}=q_{s}^{hd},h\in H,d\in D,s\in S$$ (12)

Constraints (11) and (12) respectively ensure flow conversation on railway arcs and ship arcs.

(b) *Capacity constraints*:

$$\sum_{o\in O,d\in D}q^{od}\sum_{k\in R^{od}}x_{k}^{od}\delta_{a,k}^{od}\leq b_{a},a\in A$$ (13)

$$\sum_{o\in O}q^{od}z_{hs}^{od}\leq b_{s}^{hd},h\in H,d\in D,s\in S$$ (14)

$$\sum_{o\in O,d\in D}q^{od}y_{h}^{od}\leq b_{h},h\in H$$ (15)

Constraints (13)-(14) respectively ensure that the flows on railway arc *a* and ship *s* are within the transportation capacity, while constraint (15) makes sure the flow at the hub *h* is within the transfer capacity.

(c) * Ship fulfilment chance constraint*:

$$t_{k}^{od}=\sum_{a\in A}\delta_{a,k}^{od}t_{a},o\in O,d\in D,k\in R^{od}$$ (16)

$$\mathrm{Pr}\{T_{d}^{od}+\sum_{k\in R^{od}}x_{k}^{od}t_{k}^{od}+\sum_{h\in H}y_{h}^{od}t_{h}\leq \sum_{h\in H}\sum_{s\in S}z_{hs}^{od}T_{s}^{hd}\}\geq \alpha ,o\in O,d\in D$$ (17)

Constraints (16)-(17) ensure that the cargo *od* transported by preceding railway services can catch the ensuing ship services with a possibility of at least *α*. If the cargo *od* missed the ensuing ship service, it will be viewed as an unfulfilled demand leading to a non-fulfilment penalty cost.

(d) *On-time delivery chance constraint*:

$$\mathrm{Pr}\{\sum_{h\in H}\sum_{s\in S}z_{hs}^{od}(T_{s}^{hd}+t_{s}^{hd})\leq T_{p}^{od}\}\geq \beta ,o\in O,d\in D$$ (18)

Constraint (18) ensures that the cargo *od* can arrive at the destination seaport before the promised delivery time with a possibility of at least *β*. Late arrival is allowed but leads to a penalty cost which is proportional to late delivery time.

(e) *Relationship between the decision variables and the uniqueness of the decisions*:

$$\sum_{s\in S}z_{hs}^{od}=y_{h}^{od},o\in O,d\in D,h\in H$$ (19)

$$\sum_{k\in R^{od}}x_{k}^{od}=1,o\in O,d\in D$$ (20)

$$\sum_{h\in H}\sum_{s\in S}z_{hs}^{od}=1,o\in O,d\in D$$ (21)

$$\sum_{h\in H}y_{h}^{od}=1,o\in O,d\in D$$ (22)

Constraint (19) ensure the coincidence of transfer hub selection and ship selection: a cargo transferring at hub *h* can only use those ships departing from hub *h*. Constraints (20)-(22) ensure that only one railway route, one hub and one ship can be chosen to transport each container demand.

Eqs (9)–(22) formulate a chance-constrained stochastic binary integer programming problem for the rail-sea ICRP. We will now move to the next section and explain our solution methods.

## A hybrid heuristic solution algorithm

Generally, a large-scale stochastic binary integer programming problem such as the one proposed above is NP-hard. There have been many studies dedicated to developing efficient solution methods for such problems, for example Liu [23], Pagnoncelli et al. [31], Hvattum and Løkketangen [32], Yang et al. [33], Cao et al. [34], and Wang et al [35].

In this paper, we adopt the hybrid heuristic algorithm proposed by Liu [23] to solve our proposed chance-constrained stochastic rail-sea ICRP. The algorithm is composed of three parts: (1) a *k*-shortest path algorithm for identifying the candidate routes in the railway network; (2) a stochastic simulation model (i.e. Monte-Carlo simulation) for approximating the uncertainty functions and training a neural network; and (3) a genetic algorithm for searching for the optimal solution.

The procedure of the algorithm is described as follows.

Step 1: The *k*-shortest path algorithm is employed to generate the set *R*^*od*^ of candidate routes among all feasible routes in advance. In order to avoid searching feasible routes repeatedly, a set of *k* shortest paths for each cargo is first calculated using the algorithm proposed by Yen [36].
Step 2: The Monte-Carlo simulation is first employed to generate the random input variables and joint distributions and to compute the expected value of objective function and the probability of chance constraints. Such data is used as training data to calibrate the coefficients of the neural network. The trained neural network is then employed to approximate the uncertainty functions with a high computation speed and thus improve the computational efficiency of the solution algorithm.
Step 3: Combined with the trained neural network, a genetic algorithm is used to solve the chance-constrained programming model. This combined Monte-Carlo, neural network and genetic algorithm forms the basis of the hybrid heuristic algorithm [23].

We now proceed to describe in *Section Random variables simulation* the stochastic simulation of random model variables, and in *Section Hybrid algorithm* the full hybrid solution algorithm for our proposed ICRP model.

### Random variables simulation

A Monte-Carlo method is used to simulate the random variables. Specifically, in our model, there are three types of random variables: travel time on railway arcs, travel time on ship arcs and transfer time at hubs. From these random variables, we define the following three functions which are the expected value of objective function and the two chance probabilities used in the chance constraints:

$$U_{1}:x_{k}^{od},y_{h}^{od},z_{hs}^{od}\to E\left[F(x_{k}^{od},y_{h}^{od},z_{hs}^{od})\right]$$ (23)

$$U_{2}:x_{k}^{od},y_{h}^{od},z_{hs}^{od}\to \mathrm{Pr}\{T_{d}^{od}+\sum_{k\in R^{od}}x_{k}^{od}t_{k}^{od}+\sum_{h\in H}y_{h}^{od}t_{h}\leq \sum_{h\in H}\sum_{s\in S}z_{hs}^{od}T_{s}^{hd}\}$$ (24)

$$U_{3}:z_{hs}^{od}\to \mathrm{Pr}\{\sum_{h\in H}\sum_{s\in S}z_{hs}^{od}(T_{s}^{hd}+t_{s}^{hd})\leq T_{p}^{od}\}$$ (25)

Given the values of $x_{k}^{od}$, $y_{h}^{od}$ and $z_{hs}^{od}$, the Monte-Carlo simulation is used to estimate the above three functions with stochastic travel and transfer times. Define *M* as a positive integer for the Monte-Carlo simulation and $F(x_{k}^{od},y_{h}^{od},z_{hs}^{od})$ as the objective function value. For each *m* = 1, 2, …, *M*, a sample of $t_{k}^{od}$, *t*_*h*_ and $t_{s}^{hd}$ is randomly generated according to their probability distributions and substituted in Eq (23) to get the value of $F(x_{k}^{od},y_{h}^{od},z_{hs}^{od})$, denoted as *F*_*m*_. According to the strong law of large numbers, $\sum_{m=1}^{M}F_{m}/M\to U_{1}$ as *M* → ∞. Therefore, when *M* is large enough, *U*_1_ can be estimated by $\sum_{m=1}^{M}F_{m}/M$. Similarly, for Eqs (24) and (25), *U*_2_ and *U*_3_ are approximated by *N* random samples. For each particular *n =* 1, 2, …, *N*, let *h*_1,*n*_ = 1 if $T_{d}^{od}+\sum_{k\in R^{od}}x_{k}^{od}t_{k}^{od}+\sum_{h\in H}y_{h}^{od}t_{h}\leq \sum_{h\in H}\sum_{s\in S}z_{hs}^{od}T_{s}^{hd}$ and 0 otherwise, and *h*_2,*n*_ = 1 if $\sum_{h\in H}\sum_{s\in S}z_{hs}^{od}\left(T_{s}^{hd}+t_{s}^{hd}\right)\leq T_{p}^{od}$ and 0 otherwise, then with a large integer *N*, *U*_2_ and *U*_3_ can be estimated by $\sum_{n=1}^{N}h_{1,n}/N\to U_{2}$ and $\sum_{n=1}^{N}h_{2,n}/N\to U_{3}$, respectively.

Considering the time-consuming process of stochastic simulation, a neural network is then trained and employed to approximate the uncertain functions as noted in next section.

### Hybrid algorithm

As a global search method, the genetic algorithm has a high efficiency in solving complex optimization problems. Therefore in this paper, Monte-Carlo simulation, neural network and genetic algorithm are integrated to develop a hybrid heuristic algorithm for solving the chance-constrained programming, where a genetic algorithm is used to obtain the optimal container route plan, and the Monte-Carlo simulation and neural network are used to check the feasibility of solutions and calculate the objective value by simulating the stochasticity. The details about hybrid heuristic algorithm are described in Algorithm 1.

Algorithm 1 Hybrid Heuristic Algorithm

Step 1: Initialization

        Step 1.1: Generate *k* initial feasible railway routes by Yen’s [36] algorithm for each container demand according the given container demand information and give the initial railway route set *R*^*od*^.

        Step 1.2: Generate *I* samples of decision variables as input data for training a neural network, denoted as *x*_*i*_, *y*_*i*_, *z*_*i*_ (*i =* 1, 2, …, 3000).

Step 2: Train a neural network

        Step 2.1: For each sample *x*_*i*_, *y*_*i*_, *z*_*i*_, estimate the values of uncertain functions *U*_1_, *U*_2_ and *U*_3_ as output data according to the distribution of random variables by Monte-Carlo simulation, denoted as *u*_*i*,1_, *u*_*i*,2_ and *u*_*i*,3_, respectively.

        Step 2.2: Use the input-output data *T* = {*x*_*i*_,*y*_*i*_,*z*_*i*,_*u*_*i*,1_,*u*_*i*,2_,*u*_*i*,3_|*i* = 1,…,3000} and gradient descent backpropagation algorithm to train a neural network by calibrating the values of network weights and obtain a trained neural network *N* which can then approximate the uncertain functions *u*_*i*,1_, *u*_*i*,2_, *u*_*i*,3_ by inputting *x*_*i*_, *y*_*i*_, *z*_*i*_.

Step 3: Initialize relevant parameters (initial *n =* 1) and determine *p* chromosomes (i.e. *p* initial feasible solutions of container routes) to form initial population *S* (i.e. solution set) according to the *T*. Let (x*,y*,z*) is the best solution, *E*[*F*(x*,y*,z*)] is the best objective value.

Step 4: Let *F*(*x*,*y*,*z*) is the objective function. *F*_*avg*_, *F*_max_ denote the average fitness value and the optimal fitness value in the current generation. *c* is a constant. Calculate the objective values *E*[*F*(*x*,*y*,*z*)] of all chromosomes in *S* by the trained neural network *N* and the fitness function [37]

$F^{*}\left(x,y,z\right)=\frac{\left(c-1\right)F_{avg}}{F_{\max}-F_{avg}}F\left(x,y,z\right)+\frac{F_{\max}-cF_{avg}}{F_{\max}-F_{avg}}F_{avg}$.

Step 5: Select the chromosomes by spinning the roulette wheel and update the *p* chromosomes (*x*,*y*,*z*) by crossover and mutation operations and in which the feasibility of offspring is checked by the trained neural network *N*, then *n* ← *n* + 1.

Step 6: According *T* and F*(x*,y*,z*), search the best solution (*x*^*n*^,*y*^*n*^,*z*^*n*^) in current population *S*_*n*_.

          If *E*[*F*(*x*^*n*^,*y*^*n*^,*z*^*n*^)]<*E*[*F*(x*,y*,z*)], then (x*,y*,z*)←(*x*^*n*^,*y*^*n*^,*z*^*n*^). Record and update the best solution (x*,y*,z*), the best objective value *E*[*F*(x*,y*,z*)] and the best fitness value F*(x*,y*,z*).

Step 7: If *n* > 100, output the optimal solution (x*,y*,z*) and optimal objective value *E*[*F*(x*,y*,z*)]; otherwise, go to Step 4.

**Fig 2:**
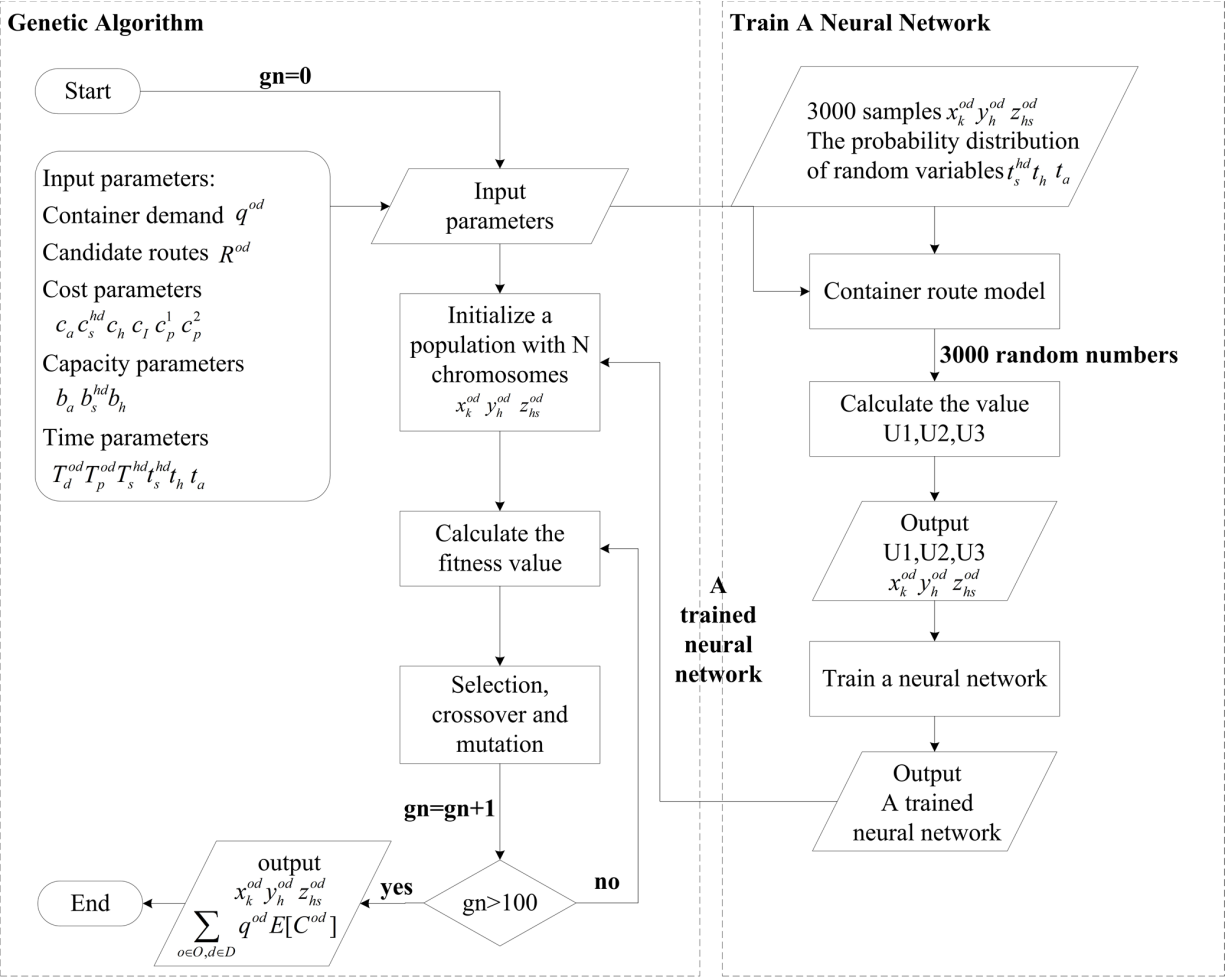
The flowchart of hybrid heuristic algorithm.

The heuristic hybrid algorithm is also illustrated as a flowchart in Fig 2. After inputting parameters and the trained neural network, a satisfied solution can be obtained in a short computation time.

## Numerical example

In this section, we present a small example to demonstrate the chance constrained model and illustrate the effect of stochastic time variables on optimal solutions and the performance of the solutions.

The following indices are used to evaluate the performance of the solutions.

(i) *Inventory cost*: the unit inventory cost multiplied by the stocking time at transfer hubs and destinations.
(ii) *Late delivery cost*: the unit delay cost multiplied by the difference of promised delivery time and actual delivery time.
(iii) *Non-fulfilment penalty cost*: the unit non-fulfilment penalty cost multiplied by the number of containers missing their ship service.
(iv) *Total cost*: the sum of total cost of all container demand base on the trained neural network.
(v) *Punctuality*: the percentage of on-time delivery.

### Numerical test

#### Case 1: A small-scale intermodal network

**Fig 3:**
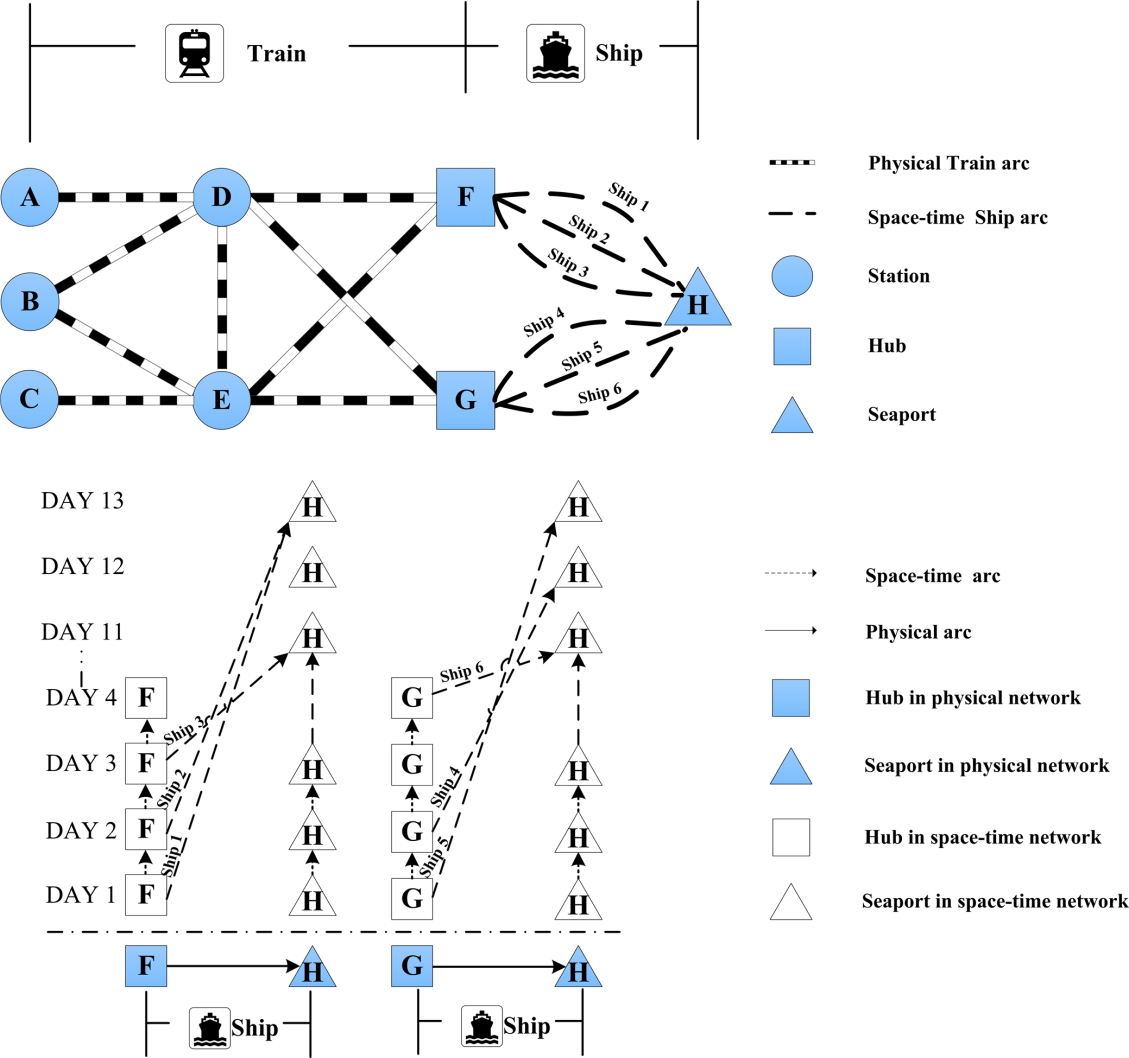
The small-scale intermodal network.

Consider a small-scale intermodal network with 5 railway stations, 2 transfer hubs and 1 destination seaport as shown in Fig 3. The cargo demand and the sea-rail network specifications are listed in Tables 3–6. All cargos are assumed to depart on time at zero hour. In addition, the travel times on all railway links and by all ships follow the same standard deviation *σ*_*a*_ and *σ*_*s*_ respectively. The cost parameters are set as follows. The late delivery cost $c_{p}^{1}$ and non-fulfilment cost $c_{p}^{2}$ are assumed to be 50 (US$/TEU/day) and 150 (US$/TEU) respectively. The unit inventory cost is set 0.8 (US$/TEU/day) according to the Regulations on Collection of Port Charges of the People’s Republic of China (MOT, 2001). In addition, we set the default confidence levels *α* and *β* as 0.9 and 0.6 respectively.

**Table 3:** Demand information in the small-scale case.

Container demand	Origin	Destination	Freight volume *q*^*od*^ (TEUs)	Delivery time $T_{p}^{od}$ (hours)
AH	A	H	68	370
BH	B	H	68	360
CH	C	H	68	350

**Table 4:** Railway information in the small-scale case.

Arc	Mean rail travel time *μ*_*a*_ (hours)	Capacity *b*_*a*_ (TEUs)	Unit cost *c*_*a*_ (US$/TEU)
(A, D)	7.5	100	120
(B, D)	8.4	130	130
(B, E)	8.4	130	130
(C, E)	7.5	130	120
(D, E)	7.5	130	120
(D, F)	7.5	130	120
(D, G)	10.6	130	157
(E, F)	10.6	130	157
(E, G)	7.5	130	120

**Table 5:** Maritime information in the small-scale case.

Candidate ship	Mean sea travel time *μ*_*s*_ (days)	Departure time $T_{s}^{hd}$ (day)	Capacity $b_{s}^{hd}$ (TEUs)	Unit cost $c_{s}^{hd}$ (US$/TEU)
Ship 1	12	1	130	190
Ship 2	11	2	140	195
Ship 3	8	3	130	205
Ship 4	10	2	130	200
Ship 5	12	1	130	190
Ship 6	7	4	140	205

**Table 6:** Transfer hub information in the small-scale case.

Transfer hub	Mean transfer time *t*_*h*_ (hours)	Capacity *b*_*h*_ (TEUs)	Unit cost *c*_*h*_ (US$/TEU)
F	5	200	50
G	6	200	50

The heuristic hybrid algorithm is coded in MATLAB R2010a, whose key parameters are provided in S1 Appendix. The program is performed on a desktop PC with a core i5 3.00GHz processor and 8GB RAM.

The case with deterministic travel time and transfer time is tested first. The optimal route for each cargo *od* demand is given as follows, leading to a total system cost of $101910:

$$\begin{array}{l} AH:A\to D\to F\to \left(Ship1\right)\to H\\ BH:B\to E\to F\to \left(Ship2\right)\to H\\ CH:C\to E\to G\to \left(Ship5\right)\to H. \end{array}$$

A stochastic case is then tested, where the multiplier *f* equals 1. The standard deviations of all rail link travel times are set equally as $\sigma_{a}=f\sigma_{a}^{0}=1hour,\forall a\in A$, the standard deviations of all ship sea travel time equally as $\sigma_{s}=f\sigma_{s}^{0}=1day,\forall s\in S$, while the range of transfer time variability at both seaports equally as (*n*_*h*_ – *m*_*h*_)/2 = 3 *hour*, ∀*h* ∈ *H*. The optimal solution is then given as follows, with a total system cost of $102260:

$$\begin{array}{l} AH:A\to D\to G\to \left(Ship6\right)\to H\\ BH:B\to D\to F\to \left(Ship2\right)\to H\\ CH:C\to E\to G\to \left(Ship5\right)\to H \end{array}$$

Comparing the results of stochastic case and deterministic case, the stochastic travel time and transfer time result in a different optimal container route plan from that under the deterministic scenario. As compared with the deterministic case, with stochasticity, the demand from origin *A* to destination *H* now transfers at seaport *G* and takes onto a ship (ship 6) that departs later, while the demand from *B* to *H* also follows a different route.

Table 7 lists the inventory cost, late delivery cost, non-fulfilment cost, total cost and punctuality for the two cases. It can be seen that the stochasticity leads to increased inventory cost, late delivery cost, non-fulfilment cost, and total cost.

**Table 7:** The cost of deterministic and stochastic parameters in the small-scale case.

Case	Inventory cost (US$)	Late delivery cost (US$)	Non-fulfilment cost (US$)	Total cost(US$)	Punctuality
Deterministic case	397	0	0	101910	100%
Stochastic case	667	100	1432	102260	97%

#### Case 2: A practical sized intermodal network

**Fig 4:**
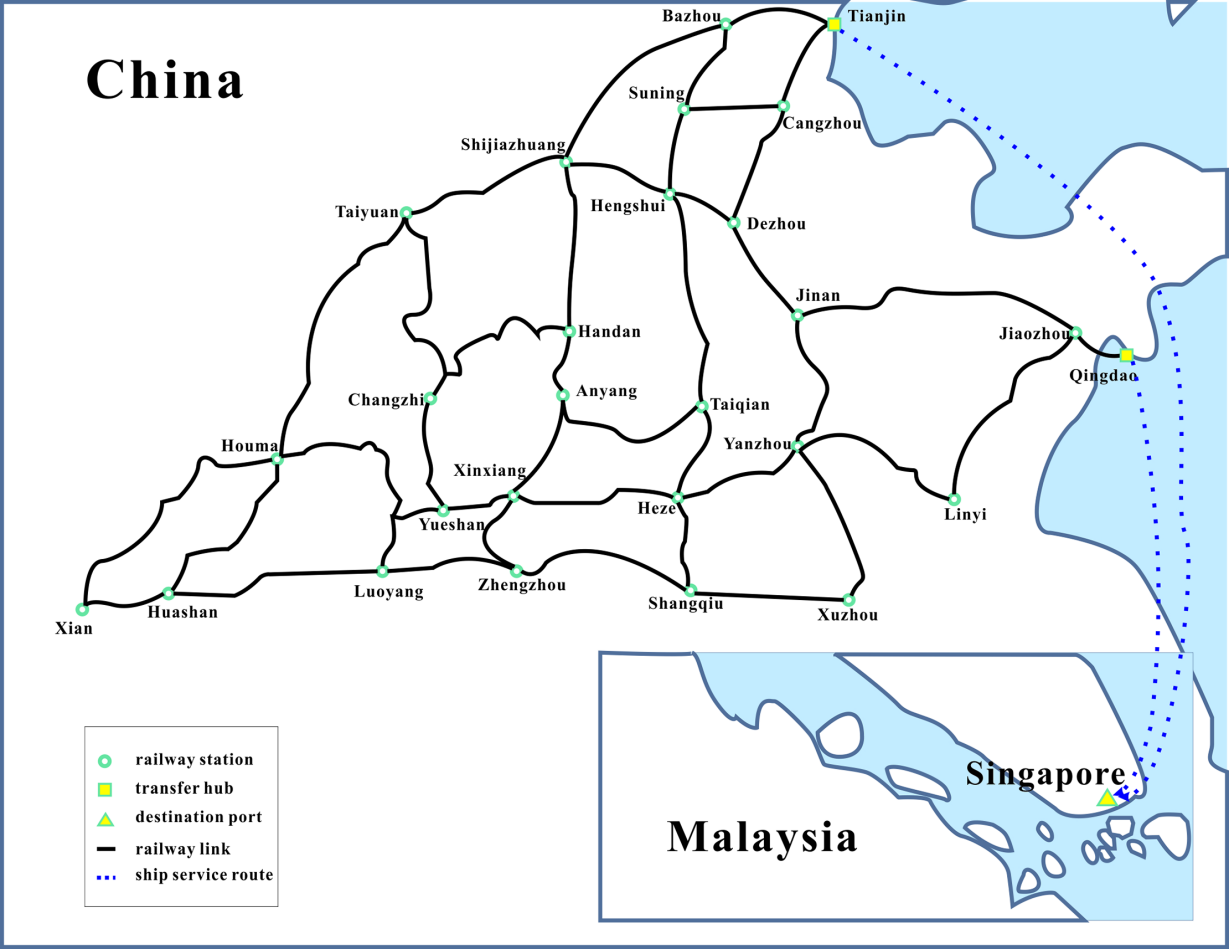
The practical sized intermodal network.

We now implement the model and algorithm on a realistic sea-rail intermodal network from China to Singapore with 25 railway stations, 2 transfer hubs and 1 destination seaport as shown in Fig 4, where six ship service routes (i.e. CSE, CISC, AEU3, AEM5, AIS, MEX2) are under operation from Tianjin and Qingdao to Singapore. It is assumed that six container demands need to be transported from China to Singapore, whose details are listed in Table 8. The transportation costs are calculated based on tariff rates provided by shipping companies and railway companies. In addition, other cost parameters are the same with those of the small-scale intermodal network.

**Table 8:** Container demand details of the practical sized case.

Container demand	Origin	Destination	Freight volume *q*^*od*^ (TEUs)	Departure time $T_{d}^{od}$ (day)	Delivery time $T_{p}^{od}$ (hours)
1	Xian	Singapore	3200	0.5	372
2	Luoyang	Singapore	2800	2.5	420
3	Zhengzhou	Singapore	2500	1.5	396
4	Taiyuan	Singapore	2000	5.5	468
5	Jinan	Singapore	2400	4.5	408
6	Xuzhou	Singapore	2600	4.5	420

The case with deterministic time parameters is tested first. Then the case with stochastic time parameters is conducted, where confidence levels *α* and *β* are 0.9 and 0.6. Besides, the standard deviations of all railway links and all ship travel time are set as *σ*_*a*_ = 1 *hour*, ∀*a* ∈ *A* and *σ*_*s*_ = 1 *day*, ∀*s* ∈ *S*, while the range of transfer time variability at both seaports equally as (*n*_*h*_ – *m*_*h*_)/2 = 3 *hour*, ∀*h* ∈ *H*. Table 9 and 10 illustrate the results of the deterministic case and stochastic case.

**Table 9:** The optimal routes in the practical sized case.

	Container demand	Optimal route
Deterministic case	1	*Xian*→*Houma*→*Taiyuan*→*Shijiazhuang*→*Bazhou*→*Tianjin*→(*ship CISC*)→*Singapore*
	2	*Luoyang*→*Zhengzhou*→*Xinxiang*→*Heze*→*Tanzhou*→*Linyi*→*Jiaozhou*→*Qingdao*→(*ship AIS*)→*Singapore*
	3	*Zhengzhou*→*Xinxiang*→*Heze*→*Yanzhou*→*Jinan*→*Jiaozhou*→*Qingdao*→(*ship AIS*)→*Singapore*
	4	*Taiyuan*→*Shijiazhuang*→*Bazhou*→*Tianjin*→(*Ship AEU3*)→*Singapore*
	5	*Jinan*→*Jiaozhou*→*Qingdao*→(*ship AEM5*)→*Singapore*
	6	*Xuzhou*→*Yanzhou*→*Jinan*→*Dezhou*→*Cangzhou*→*Tianjin*→(*ship AEU3*) →*Singapore*
Stochastic case	1	*Xian*→*Houma*→*Taiyuan*→*Shijiazhuang*→*Bazhou*→*Tianjin*→(*ship CSE*) →*Singapore*
	2	*Luoyang*→*Yueshan*→*Xinxiang*→*Anyang*→*Handan*→*Shijiazhuang*→*Bazhou*→*Tianjin*→(*ship AEU3*)→*Singapore*
	3	*Zhengzhou*→*Xinxiang*→*Anyang*→*Handan*→*Shijiazhuang*→*Hengshui*→*Dezhou*→*Cangzhou*→*Tianjin*→(*ship CISC*)→*Singapore*
	4	*Tiayuan*→*Shijiazhuang*→*Hengshui*→*Suning*→*Bazhou*→*Tianjin*→(*ship CISC*)→*Singapore*
	5	*Jinan*→*Jiaozhou*→*Qingdao*→(*ship AIS*)→*Singapore*
	6	*Xuzhou*→*Yanzhou*→*Linyi*→*Jiaozhou*→*Qingdao*→(*ship AIS*)→*Singapore*

**Table 10:** The cost of deterministic and stochastic cases in the practical sized case.

Case	Inventory cost (US$)	Late delivery cost (US$)	Non-fulfilment cost (US$)	Total cost(US$)	Punctuality
Deterministic case	3.8×10^4^	0	0	1.4×10^7^	100%
Stochastic case	6.1×10^4^	1.5×10^6^	2.3×10^4^	1.5×10^7^	73%

As the results presented in Table 10 show, the optimal route is strongly affected by the stochastic travel time and transfer time. For the container demand from Zhengzhou to Singapore, the railway route, transfer hub and ship route all change in order to reduce high penalty cost and satisfy on-time delivery request. With respect to the relevant costs, the inventory cost, late delivery cost and non-fulfilment cost all increase due to the delay caused by stochasticity. In addition, the punctuality also falls from 100% to 73%, leading to a lower service level.

### Sensitivity analysis

To study the influence of stochastic variables and model parameters, the tests under different standard deviations of travel time and different confidence level for the small-scale intermodal network are conducted.

#### The impact of travel time variability

In this section, we investigate the impact of rail link and sea link travel times on the optimal solutions of our stochastic ICRP for the small-scale case. The optimal solutions and the performance of them under different multiplier *f* (i.e. different travel time variabilities *σ*_*a*_ and *σ*_*s*_) are calculated and shown in Table 11.

**Table 11:** The optimal route plans with different multiplier *f* in the small-scale case.

Scenario	Multiplier *f*	Inventory cost (US$)	Late delivery cost (US$)	Non-fulfilment cost (US$)	Total cost (US$)	Punctuality	Optimal routes
1	0 (deterministic case)	397	0	0	101910	100%	*AH*:*A*→*D*→*F*→(*Ship* 1) →*H*
							*BH*:*B*→*E*→*F*→(*Ship* 2) →*H*
							*CH*:*C*→*E*→*G*→(*Ship* 5) →*H*
2	1	667	100	1432	102260	97%	*AH*:*A*→*D*→*G*→(*Ship* 6) →*H*
							*BH*:*B*→*D*→*F*→(*Ship* 2) →*H*
							*CH*:*C*→*E*→*G*→(*Ship* 5) →*H*
3	2	723	1297	0	103190	88%	*AH*:*A*→*D*→*F*→(*Ship* 2) →*H*
							*BH*:*B*→*E*→*G*→(*Ship* 6) →*H*
							*CH*:*C*→*E*→*F*→(*Ship* 2) →*H*
4	3	1123	1331	0	105710	90%	*AH*:*A*→*D*→*G*→(*Ship* 6) →*H*
							*BH*:*B*→*D*→*F*→(*Ship* 3) →*H*
							*CH*:*C*→*E*→*G*→(*Ship* 6) →*H*
5	4	979	4166	52	108550	81%	*AH*:*A*→*D*→*F*→(*Ship* 3) →*H*
							*BH*:*B*→*D*→*G*→(*Ship* 6) →*H*
							*CH*:*C*→*E*→*G*→(*Ship* 4) →*H*
6	5	1046	7121	0	111070	76%	*AH*:*A*→*D*→*G*→(*Ship* 6) →*H*
							*BH*:*B*→*D*→*F*→(*Ship* 3) →*H*
							*CH*:*C*→*E*→*G*→(*Ship* 4) →*H*
7	6	1087	10192	160	114800	72%	*AH*:*A*→*D*→*F*→(*Ship* 3) →*H*
							*BH*:*B*→*D*→*G*→(*Ship* 4) →*H*
							*CH*:*C*→*E*→*G*→(*Ship* 6) →*H*

As shown in Table 11, the late delivery cost and total cost go up obviously with the growth of value *f*, while the punctuality presents an opposite trend that the higher the value *f*, the lower the punctuality. This implies higher travel time variability not only has an effect on route plans but results in more operation cost and lower service level. In addition, inventory cost rises to 1087 US dollars with some fluctuation, while the non-fulfilment cost changes irregularly.

#### The impact of chance confidence levels

Our proposed stochastic ICRP model includes two confidence levels: the on-time probability *α* for the chosen ship service, and the other probability *β* for on-time delivery at the final destination. In this section, we investigate the effects of these two probabilities on the optimal solutions. For the first probability *α*, the small-scale case with different values of *α* is tested. We found, due to high penalty cost of non-fulfilment fixed for this case study, the route plan with high possibility of fulfilment is always favoured as the optimal one. Therefore changes in the fulfilment confidence level *α* do not yield significantly different results. However, the on-time delivery confidence levels *β* are shown to have a significant impact on the optimal solutions.

**Fig 5:**
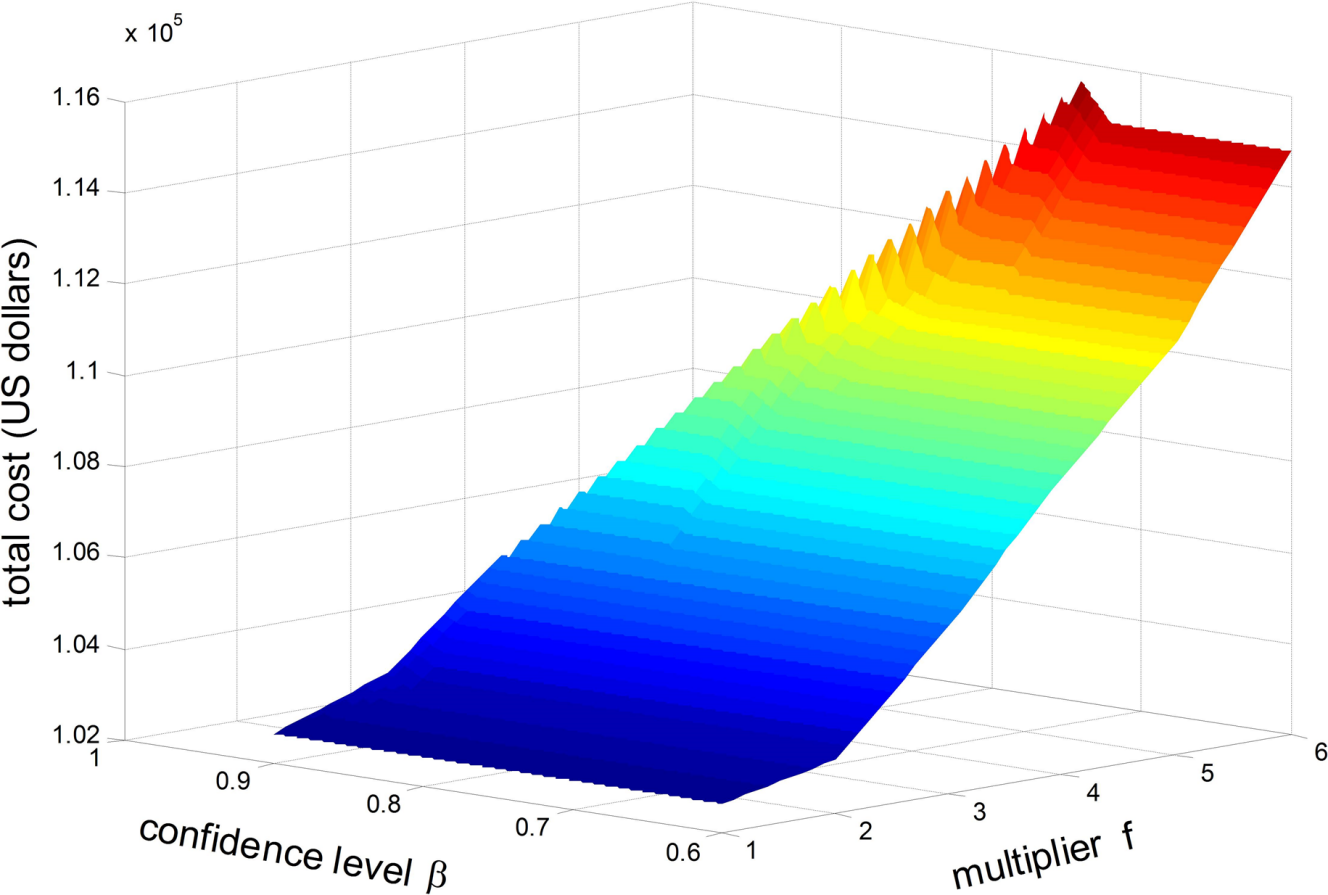
The total cost with different confidence level *β* and multiplier *f*.

Fig 5 shows the total costs of optimal solutions under different values of *β* and different values of *f* (i.e. different travel time variabilities *σ*_*a*_, *σ*_*s*_). The fulfilment probability *α* in this test is 0.9. It can be seen that, as the value *f* grows, the total cost also increases under the same confidence level *β*, which is consistent with what we discussed in Table 11. This indicates that, with the increase of travel time variabilities, higher operation cost is required to remain the service level, i.e. the possibility for on-time delivery.

Similarly, with the increase of *β*, the total cost goes up under the same value *f*. This implies that, with high confidence level *β*, the minimum-cost solution can not satisfy the request of on-time delivery probability. Consequently, it leads the change of optimal routes and higher operation costs in order to achieve higher punctuality.

## Conclusion

In this paper, the intermodal container routing problem (ICRP) with stochastic time variables is formulated as a binary integer chance-constrained programming model to minimise total cost in a sea-rail transportation network. A hybrid heuristic algorithm incorporating Monte-Carlo, neural network and genetic algorithm is proposed to solve the problem under pre-determined confidence levels of chance constraints. A numerical example is presented to demonstrate the validity of the proposed model in solving ICRP. Sensitivity tests are conducted to examine the influence of model variables on the total cost and optimal routes for each demand.

The results confirm that transportation stochasticity can lead to different optimal routing plans compared to the deterministic case. In addition, the stochastic travel time and transfer time not only increase inventory cost and total cost, but also incur the late delivery cost and non-fulfilment cost. The costs increase with increasing travel time variability. With higher on-time delivery probability, the number of feasible solutions reduces. This implies that higher operation costs would be required to meet higher service requirements.

For further research, as in Meng et al.[38], the demand could also be stochastic. Therefore we can further include the variation of demands in the container routing problem. Meanwhile, as stochastic demand and stochastic travel time can both affect the container route choices and the total operation cost, which conversely affects the performance of the intermodal network, it is therefore meaningful to integrate the container routing choice in the intermodal network design process.

## Supporting information

S1 Appendix Parameter setting for the numerical example. (DOCX)
