# Supplementary material for: A chance-constrained stochastic approach to intermodal container routing problems
Source: PLoS One. 2018 Feb 13;13(2):e0192275. doi: 10.1371/journal.pone.0192275 (PMC5811005; doi:10.1371/journal.pone.0192275)
Supplement: S1 Appendix — (DOCX) [file pone.0192275.s001.docx]

# Appendix Parameter setting for the numerical example

The key parameters of heuristic hybrid algorithm are shown in Table 12 and 13. Furthermore, for GA, the fitness function $F^{*}\left(x\right)$ based on the linear scaling method is employed to evaluate each chromosome, given in Eq. (26),

$F^{*}(x)=\frac{(c-1)F_{avg}}{F_{\max}-F_{avg}}F(x)\text{+}\frac{F_{\max}-cF_{avg}}{F_{\max}-F_{avg}}F_{avg}$ (26)

where $F\left(x\right)$ is the objective function, and $F_{avg}$ and $F_{\max}$ respectively denote the average fitness value and the optimal fitness value in the current generation. *c* is a constant set to be 2 in our case.

Table 12. Genetic algorithm parameters.

| Population size | 30 |
| --- | --- |
| The probability of crossover | 0.8 |
| The probability of mutation | 0.5 |
| Stopping criterion | The number of generation |
| Evaluation function | Linear scaling method |
| Selecting operation | Roulette Wheel Selection |
| Crossover operation | Multi-point crossover |
| Mutation operation | Bit string mutation |

Table 13. Neural network parameters.

| Epoch | Mean squared error | Neural network structure |
| --- | --- | --- |
| 10000 | 10^ (-6) | 3 input neurons, 30 hidden neurons and 6 output neurons |
| Hidden layer  Activation function | sigmoid function: Linear function: $\sigma_{1}(x)=\frac{1}{1+e^{-x}}$ | |
| Output layer  Activation function | Linear function: $\sigma_{2}(x)=x$ | |
| Training algorithm | Gradient descent backpropagation with adaptive learning rate | |
